# NSAIDs Use and Reduced Metastasis in Cancer Patients: results from a meta-analysis

**DOI:** 10.1038/s41598-017-01644-0

**Published:** 2017-05-12

**Authors:** Xiaoping Zhao, Zhi Xu, Haoseng Li

**Affiliations:** 1 Center of Stomatology, Tongji Hospital, Tongji Medical College, Huazhong University of Science and Technology, Wuhan, China; 2 Department of Stomatology, Union Hospital, Tongji Medical College, Huazhong University of Science and Technology, Wuhan, 430022 China

## Abstract

This meta-analysis investigated the relationship between non-steroidal anti-inflammatory drugs (NSAIDs) and lymph node/distant metastasis. Relevant sources were identified from MEDLINE, EMBASE, PubMed, and Cochrane Library. Studies that reported the odds ratio (OR)/risk ratio (RR)/hazard ratio (HR) with 95% confidence intervals (CIs) for the associations of interested outcomes were included. Pooled effect estimates were obtained by using random- or fixed-effect model depending on the heterogeneity across these studies. Sixteen studies involving 202780 participants, including prostate, breast, lung, and colorectal cancer patients, were included. Compared with the reference, generally patients exposed to NSAIDs at pre- and post-diagnosis experienced a significantly reduced risk of distant metastasis (RR 0.708, 95% CI 0.586–0.856 and RR: 0.484, 95% CI: 0.393–0.595, respectively), including prostate cancer (pre-diagnostic use: RR = 0.874, 95% CI, 0.787–0.97; post-diagnostic use: RR = 0.482, 95% CI 0.359–0.647), and breast cancer (pre-diagnostic use: RR = 0.644, 95% CI 0.565–0.735; post-diagnostic use: RR = 0.485, 95% CI 0.362–0.651). However, lymph node metastasis was weakly related with pre-diagnostic use of NSAIDs (RR = 0.949, 95% CI 0.914–0.985). NSAIDs are related to a significantly reduced risk of metastasis development, regardless of pre-diagnostic or post-diagnostic use. However, NSAIDs and lymph node metastasis are weakly associated. Our finding suggested a novel metastasis management.

## Introduction

Non-steroidal anti-inflammatory drugs (NSAIDs) are widely prescribed for patients with coronary heart disease and rheumatoid arthritis. Currently, NSAIDs are becoming necessary in management of cancer patients. One important reason for the use of NSAIDs is that blood coagulation system is often activated in the course of malignancies, and the risk of venous thromboembolism increases with locally advanced or metastatic cancer. Recently, NSAIDs are further recommended as the primary drug for prevention of colorectal cancer^1^.

Numerous fundamental studies and clinical trials have focused on the negative correlation between NSAIDs and patients’ overall mortality. The protective effect of NSAIDs is widely hypothesized to be attributed to the inhibition of cancer metastasis. However, the lines of evidence supporting the potential of NSAIDs to reduce cancer metastasis are conflicting^2–4^. While some studies found no significant association between NSAID use and cancer metastasis, other studies have demonstrated that NSAIDs are associated with reduced risk of metastasis and even with reduced cancer incidence. Interpretation of this conflicting findings has been complicated by different baseline characteristics of study populations (e.g., men vs. women; aspirin vs. warfarin vs. heparin; pre-diagnostic use vs. post-diagnostic use vs. non-use of NSAIDs; regular use vs. frequent use vs. non-use; lymph node (LN) metastasis vs. distant metastasis; breast cancer vs. lung cancer vs. prostate cancer; early-stage cancer vs. late-stage cancer; long-term follow-up vs. short-term follow-up, and different types of studies (case-control studies vs. cohort studies vs. random-controlled trials (RCT)). To integrate metastatic theory with clinical treatment, as well as provide an indication for treatment with anticoagulant agents to physicians and cancer patients, we conducted a meta-analysis on the impact of pre-diagnostic or post-diagnostic use of NSAIDs on cancer with LN or distant metastasis.

## Results

### Study Selection and Characteristics

**Figure 1:**
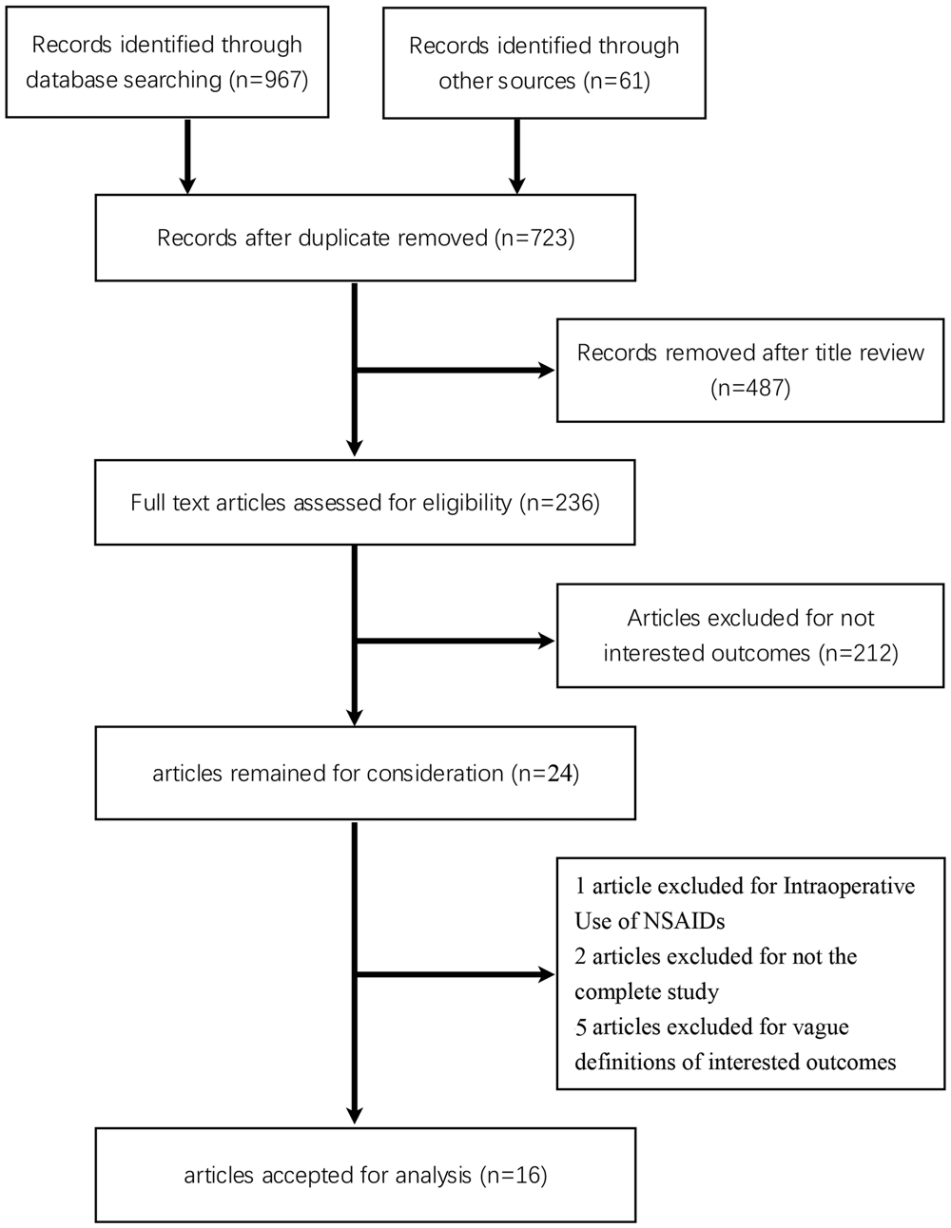
Flow diagram of study selection.

A total of 962 relevant articles were initially retrieved through literature search engine. Following omissions of duplicated articles and reports without interested outcome, 24 studies were collected for consideration. After full text review, 16 studies were selected for this meta-analysis^5–28^ (Fig. 1, Supplementary Dataset 8).

**Figure 2:**
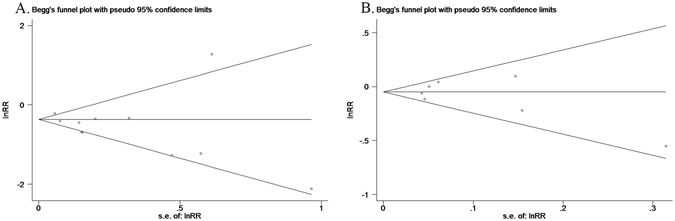
Public bias assessment of included studies. (**A**) Funnel plots showing association between distant metastasis and NSAID use of patients. (**B**) Funnel plots showing association of lymph node metastasis with NSAID use of patients.

Next, the quality of selected studies was evaluated according to Newcastle-Ottawa Scale (NOS) criterion. The accumulated stars of included case–control studies ranged from 7 to 8, and cohort studies’ score ranged from 7 to 9. Therefore, we considered the selected studies were high-quality studies (Supplementary Dataset 9). Besides, Results from Begg’s, Egger’s and funnel plot’s asymmetry tests all showed that there is no evidence of publication bias in both distant metastasis group and lymph metastasis group (Fig. 2).

A total of 202780 patients and at least 45044 events were involved in the 16 eligible studies, including 1 RCT, 11 cohort studies, and 4 case reports. The sizes of these studies ranged from 74 to 78615. Of these primary trails, all had described independent, continuous sampling of their cohort. A total of 10 studies were conducted in USA, 2 in Sweden, and the remaining 4 studies in UK, Canada, Ireland, and Italy. Among these studies, aspirin was the most predominant NSAID given to patients. These studies have investigated many cancers, including breast cancer (7 studies), prostate cancer (5 studies), colorectal cancer (1 studies), and esophageal cancer (1 study). Moreover, multi-cancers were found in two studies. A total of 10 trials have investigated the association between NSAIDs and distant metastasis, 5 studies explored the connection between NSAIDs and LN metastasis, and one study determined the simultaneous change in distant metastasis and LN metastasis in patients exposed to NSAIDs. Apart from the four trails that have explored the impact of post-diagnostic use of NSAIDs on distant metastasis, the remaining 12 studies investigated the effect of pre-diagnostic use of NSAIDs on distant/LN metastasis. Adjusted HRs/RRs/ORs could be determined in all studies, with 12 studies reporting adjusted estimates (Supplementary Dataset 9).

### Association between NSAIDs and Cancer Distant Metastasis

Eleven studies involving 247,826 patients and more than 40,000 events reported on the risk estimates for cancer distant metastasis in patients exposed to NSAIDs before or after diagnosis. Among the eleven studies, ten studies reported the negative association between NSAIDs and cancer metastasis, although the results from two studies were not statistically significant. The remained study found positive relationship between NSAIDs and cancer metastasis with statistically significance. Overall, compared with the reference group, cancer patients taking NSAIDs showed a significantly reduced risk for metastasis development (RR: 0.623, 95% CI 0.515–0.753, p < 0.001) (Supplementary Dataset 1).

A considerable heterogeneity of RRs was observed across the Eleven studies (*I*^2^ = 69.5%). We subsequently stratified these studies based on exposure to NSAIDs. Moderate heterogeneity was detected in the pre-diagnostic NSAID use group (RR: 0.708, 95% CI 0.586–0.856, p < 0.001, *I*^2^ = 63.4%), whereas no heterogeneity was detected among trials that employed post-diagnostic use of NSAID (RR: 0.484, 95% CI 0.393–0.595, *I*^2^ = 0.0%) (Supplementary Dataset 1). Therefore, a sensitivity analysis was performed to detect the study that have mainly contributed to this high heterogeneity. After omitting the study of James L. Araujo, the heterogeneity of pre-diagnosis NSAID use group decreased (*I*^2^ = 48.3%) and the inhibition by pre-diagnostic NSAID use slightly increased (RR 0.729, 95% CI 0.671–0.791, p < 0.001), although the overall heterogeneity of the studies remained at moderate level (*I*^2^ = 64.8%) (Supplementary Dataset 2).

We subsequently investigated the correlation between NSAID use and the indicated cancer types. In prostate cancer, the summarized results suggested a negative association between NSAIDs and prostate cancer metastasis with a considerable heterogeneity (RR = 0.817, 95% CI 0.74–00.902, p = 0.006, *I*^2^ = 70.9%). When we stratified these studies based on the time of exposure to NSAIDs, the risk of metastasis development both in pre-diagnostic and post-diagnostic NSAID use groups decreased (pre-diagnosis: RR = 0.874, 95% CI 0.787–0.97, p = 0.012; post-diagnosis: RR = 0.482, 95% CI 0.359–0.647, p < 0.001); moreover, heterogeneity was not detected in both groups (pre-diagnosis: *I*^2^ = 0.00%; post-diagnosis: *I*^2^ = 0.00%) (Supplementary Dataset 3). Similar results were obtained in trials that have studied breast cancer (overall use: RR = 0.615, 95% CI 0.546–0.693, p < 0.001, *I*^2^ = 29.3%; pre-diagnostic use: RR = 0.644, 95% CI 0.565–0.735, p < 0.001, *I*^2^ = 0.00%; post-diagnostic use: RR = 0.485, 95% CI 0.362–0.651, p < 0.001, *I*^2^ = 53.4%) (Supplementary Dataset 4). For both lung cancer and colorectal cancer, the pooled RR is consistently less than 1 with a high heterogeneity (Supplementary Dataset 5).

### Association between NSAIDs and Cancer LN Metastasis

We subsequently investigated the relationship between NSAIDs and lymph node metastasis. Six studies involving 110735 participants and approximately 20,000 patients with LN metastasis were recruited in this analysis. All of these studies (predominantly breast cancer and prostate cancer clinical trials) focused on the relationship between pre-diagnostic use of NSAIDs and LN metastasis. Four of these studies found an association between pre-diagnosis use of NSAIDs and a reduced risk of LN metastasis, although only one study reported statistically significant results. However, compared with the referent group, the risk of LN metastasis slightly decreased in the pre-diagnostic NSAID use group (RR = 0.949, 95% CI 0.914–0.985, p = 0.006, *I*^2^ = 36.3%) (Supplementary Dataset 6).

In these trails, breast cancer was involved in 5 studies, prostate cancer in 2 studies, colorectal cancer in 1 studies, and lung cancer in 1 study. Therefore, we performed a meta-analysis to determine the relationship between NSAIDs and breast cancer LN metastasis. A negative relationship between NSAIDs and LN metastasis was detected in the summarized studies, although the results were not statically significant (RR = 0.958, 95% CI 0.913–1.004, p = 0.075, *I*^2^ = 34.8) (Supplementary Dataset 7).

## Discussion and Conclusion

Metastasis is a multi-sequential process accounting for most cancer-related deaths. Although the mechanisms regulating cancer metastasis remain incompletely investigated, metastasis is found to be inherently inefficient^29,30^. Even after successful invasion of blood vessels, most cancer cells become dormant because of factors such as anoikis, fluid shear stresses, and immunological surveillance. Therefore, the use of an appropriate intervention for cancer metastasis is a promising strategy to improve the prognosis of cancer patients.

NSAIDs are non-selective or selective COX-1/2 inhibitors, which are wildly prescribed for pain killing, fever reduction, and even anti-inflammation. Nowadays, NSAIDs are used to prevent DVT in cancer patients. Accumulated lines of evidence have confirmed the safety of long-term use of low-dose NSAIDs, the positive association between NSAIDs and improved prognosis^31–33^, and the negative association between NSAIDs and carcinogenesis. The reduced mortality of cancer patients exposed to NSAIDs has attracted great attention. Actually, some recent studies have addressed the potential of NSAIDs to inhibit cancer metastasis: study from Huang *et al*. suggested post-diagnosis NSAIDs use was inversely associated with metastasis^2^; Elwood *et al*.’s research revealed NSAIDs use reduced metastatic spread^3^; Algra *et al*. suggested regular use of aspirin was correlated with cancers with distant metastasis^34^. Although all the studies have suggested the benefits of NSAIDs use in cancer patients, some defects may complicate the interpretation of the association. Firstly, the criteria of articles selection are inconsistent among these studies. For example, the data of local and regional relapse is mixed with lymph node metastasis, even distant metastasis. More importantly, the number of included trials is limited and there is a considerable heterogeneity among these studies. However, the interesting relation between NSAIDs and cancer metastasis revealed by these studies urges us to define the place of NSAIDs in cancer metastasis.

The present meta-analysis, which involves approximately 300,000 participants from 16 studies, has confirmed the antineoplastic effects of NSAIDs in multi-cancer metastasis, regardless of whether NSAIDs is used at pre-diagnosis or post-diagnosis. In terms of LN metastasis, weak inhibition was observed in cancer patients exposed to NSAIDs at pre-diagnosis. Our results have suggested the underappreciated role of NSAIDs in cancer metastasis.

The results showed that both pre- and post-diagnostic use of NSAIDs were related to reduced distant metastasis. However, high heterogeneity was detected in the trials that employed pre-diagnostic NSAID use. The heterogeneity among the pooled studies decreased after omitting the trial performed by James L. Araujo, which we then reviewed. James L. Araujo reported that increased risk of cancer metastatic development was found in patients with pre-diagnosis exposure to NSAIDs, contrary to the conclusion of other studies. Although no defect was found in the study design and in patient enrollment, some flaws may have contributed to the inconsistent results. First, the number of patients involved in this trial was small and the detailed information on NSAID use was subsequently omitted in this study. For example, patients involved in this trial may have intermittently taken NSAIDs, possibly contributing to the confounding results^15,35^. Moreover, to our best knowledge, this study is the only work that focused on the relationship between NSAIDs and esophageal cancer metastasis; therefore, other related information is unavailable. However, multiple studies have demonstrated that NSAID use is associated with reduced incidence of esophageal cancer and reduced esophageal cancer-related mortality, also inconsistent with the conclusion of this study^36,37^. Overall, no conclusion can demonstrate the relationship between NSAIDs and esophageal cancer metastasis until further well-designed studies are performed.

The underlying mechanisms involved in the association between NSAIDs and distant metastasis inhibition remains incompletely investigated. One possible explanation is that NSAIDs inhibit COX2^38^. Abnormally high COX2 expression was observed in multi-cancers. Disordered COX2/PGE pathway is involved in multi-cancer processes, including carcinogenesis, proliferation, and metastatic spread; additionally, inhibition of COX2/PGE pathway with NSAIDs can restrain cancer cell lines and xenograft models. Mutual promotion relationship between cancer metastasis and cancer-associated thrombosis is possibly another one of the underlying mechanisms. Abnormally high constitutive level of tissue factor (TF), one key regulator of hemostasis, is expressed by metastatic cancer cells, cancer microparticles, and cancer-associated monocytes and macrophages; TF can promote thrombosis formation by activating the extrinsic pathway of coagulation cascade^39^; moreover, activated blood coagulation system promotes platelet aggregation, which shields cancer cells in multi-dimensions, including prevention of exposure of tumor cells to NK cells, inhibition of maturation of dendritic cells, promotion of camouflaging of cancer cells as normal cells, reduction of fluid shear stresses by the surrounding platelet, and promotion of cancer cell survival through a COX2-dependent pathway against anoikis. Furthermore, inflammation induced by thrombosis could result in endothelial damage, which may further augment the thrombosis against the anticoagulated system and result in vascular leak, facilitating the escape of cancer cells from blood vessels. Consequently, NSAIDs may disrupt the relationship between cancer metastasis and cancer-associated thrombosis via suppression of platelet function, which is detrimental for the disseminated cancer cells in the bloodstream. Finally, reconstruction of pre-metastasis niche can contribute to metastasis inhibition. By inhibiting the chemotaxis of leukocytes and by restraining the release of bradykinin and aggression of thrombocyte inhibition, NSIADs could suppress inflammation. More importantly, inflammation is one driving force accelerating cancer metastasis^40^. Therefore, NSAIDs may further inhibit the formation metastasis foci by suppressing inflammation of pre-metastatic niche and then transforming the prepared microenvironment into adverse microenvironment for the coming cancer cells.

Noteworthily, inhibition of metastasis in patients with pre-diagnostic exposure to NSAIDs is inconsistent with the conclusions of other clinical studies or meta-analyses^2^. Further analysis of our studies have shown the collective long-term pre-diagnostic use of NSAIDs in these studies (the majority patients used NSAIDs for >1 year, data not shown), although NSAID dosage varied. The prolonged exposure to NSAIDs is possibly one important factor contributing to distant metastasis inhibition given that dissemination of cancer cells is not related to cancer size, and metastasis may occur in an early stage of cancer^41,42^. As mentioned above, by preventing the change in microenvironment during cancer metastasis and pre-metastatic niche, long term pre-diagnostic use of NSAIDs could activate the immune system to kill cancer cells in the blood stream and pre-metastatic niche.

LN metastasis, the most common mechanism of cancer spread, indicates poor prognosis of cancer patients. Although obvious effect of NSAIDs on cancer distant metastasis is observed in many studies, LN metastasis was only weakly inhibited in patients treated with NSAIDs. This finding may be partially attributed to the different mutated genes, disorganized signal pathways, and mechanisms mediating cancer with LN and distant metastases. For example, the antiplatelet property of NSAIDs is widely speculated to contribute to cancer inhibition. However, platelets are absent in the lymphatic system, possibly rendering NSAIDs nonfunctional in the lymphatic system. Moreover, another important factor was the small number of studies acquired in the analysis (multi-cancer: 1 study; prostate cancer: 1 study; breast cancer: 4 studies; lung cancer: 0 study; colorectal cancer: 0 study). This insufficient information significantly limited the determination of the effect of NSAIDs on LN metastasis. Therefore, more well-designed fundamental and clinical studies are warranted to investigate the relationship between NSAIDs and LN metastasis.

Strengths of this meta-analysis lies in its strict inclusion criteria, large number of studies and patients analyzed, and the robustness of the findings in sensitivity analyses. However, our study encountered several limitations. First, the clear and understandable discrepancy in sample sizes lies among the randomized, cohort, and case control studies. Second, different types of studies were included in the primary analysis; heterogeneity of effect magnitude (RR/OR/HR) among studies is anticipated. However, when we performed stratified analyses based on the indicated cancer and the time of NSAID prescription, the heterogeneity among studies became slightly, and pooled RRs were consistently lower than 1. Moreover, even the intraoperative use of NSAIDs may reduce cancer distant metastasis. All of these lines of evidence have suggested the negative correlation between NSAIDs and cancer metastasis. Third, insufficient studies on the indicated cancers were collected in this study. For example, only two and three studies have investigated the pre-diagnostic use of NSAIDS in colorectal cancer and breast cancer with distant metastasis, respectively. However, heterogeneity was obvious (colorectal cancer: *I*^2^ = 78.8%; breast cancer: *I*^2^ = 53.4%); moreover, the cohort study on esophageal cancer showed that pre-diagnosis use of NSAIDs increases the risk of cancer metastasis, although many analyses have demonstrated the negative relationship between NSAID use and patient mortality. Therefore, this insufficient information limits the analysis of the effect of pre-diagnostic and post-diagnostic use of NSAIDs in indicated cancers, although the results of the pooled analysis consistently showed statistical significance. Fourth, the lack of information on the side effects of NSAIDs, subtype of cancers, and patients’ exposure to etiological factors and on the inconsistent spice, dose, and term of usage of NSAIDs, and the varied term of follow up may collectively cause confusion on the use of NSAIDs in management of cancer. Fifth, like all meta-analyses, our study is limited by its being a retrospective analysis.

In conclusion, the results of this meta-analysis suggest the potential of NSAIDs in the management of cancer metastasis, regardless of whether NSAIDs are used at pre-diagnosis or post-diagnosis. However, the acquired information is insufficient to support the recommendation or use of NSAIDs in management of indicated cancer until further large well-designed trials, such as RCTs, are completed. In light of our finding and the confusion in the management of cancer metastasis, our results highlight a novel strategy to manage cancer metastasis.

## Method

### Search Strategy

Based on PRISMA guidelines, this meta-analysis of published studies was performed without language restrictions (Supplementary Dataset 9). Relevant studies were extracted from electronic databases, including MEDLINE (published in PubMed from January 1, 1946 to December 1, 2016), EMBASE (January 1, 1980 to December 1, 2016), PubMed (January 1, 1980 to December 1, 2016), and Cochrane Library (January 1, 1980 to December 1, 2016). The combined text and MeSH heading search strategy included “Anti-Inflammatory Agents,” Non-Steroidal,” “cyclooxygenase 2 inhibitor,” “Neoplasms,” “lymph node metastasis,” and “distant metastasis”. Furthermore, the references of all relevant articles were manually scrutinized to supplement our search.

### Study Selection

Relevant studies were considered acceptable based on the following criteria: (1) they were RCTs, cohort studies, or case-control studies (cell lines and animal studies, cross-sectional studies, reviews, meetings, commentaries, and letters were excluded); (2) NSAIDs were taken before or after diagnosis, and the outcomes of interest included LN metastasis and distant metastasis; (3) effect magnitude, including odds ratio (OR), risk ratio (RR), and hazard ratio (HR) and the corresponding 95% confidence interval (CI) (or data used to calculate the effect magnitude) were reported; and (4) studies were independent. When multiple studies based on the same trials were identified, only the most recent or complete study was included.

### Methodological Quality Assessment and Data Extraction

Non-randomized studies (MINORS) were evaluated according to the Newcastle–Ottawa Scale (NOS) criteria, and RCT study was assessed using the Cochrane Collaboration’s tool for assessing risk of bias. Disagreements on the quality evaluation of the include papers were resolved by comprehensive discussion. In this analysis, studies awarded seven or more were taken as high-quality studies^43^.

Three authors (ZXP, XZ, and LHS) extracted the following information from each study: study characteristics (study design, first author’s name, publication year, country, patients, and numbers of events), participants’ characteristics (mean age and gender), drug characteristics (species, dosage, and term of usage), and analysis strategy (statistical models, confounders adjusted for, effect sizes, and 95% CIs) (Supplementary Dataset 9).

### Publication bias Assessment

Latent publication bias was determined by Begg’s adjusted rank correlation test and Egger’s regression asymmetry tests, and also by inspection for a funnel plot’ asymmetry tests.

### Statistical Analysis

RRs were taken as the common measure of association across studies. Summary RRs were estimated by pooling study-specific estimates using random- or fixed-effect models according to the heterogeneity among studies. The *I*^2^ statistic was calculated to assess the heterogeneity of reported RRs in all studies^44^. Basically, low, moderate, and high heterogeneity were defined as *I*^2^ of <50%, 50–75%, and >75%, respectively. Sensitivity analysis was performed to assess the effects of the quality of the selected study. All analyses were performed using Stata version 14.0 (Stata Corp), and a p value of <0.05 indicated statistical significance.

## Electronic supplementary material

Supplementary Dataset 9

Supplementary Dataset 1

Supplementary Dataset 2

Supplementary Dataset 3

Supplementary Dataset 4

Supplementary Dataset 5

Supplementary Dataset 6

Supplementary Dataset 7

Table legends

Supplementary Dataset 8

## References

[1] Bibbins-Domingo K, Force USPST (2016). Aspirin Use for the Primary Prevention of Cardiovascular Disease and Colorectal Cancer: U.S. Preventive Services Task Force Recommendation Statement. Ann Intern Med.

[2] Huang XZ (2015). Aspirin and nonsteroidal anti-inflammatory drugs after but not before diagnosis are associated with improved breast cancer survival: a meta-analysis. Cancer causes & control: CCC.

[3] Elwood PC (2016). Aspirin in the Treatment of Cancer: Reductions in Metastatic Spread and in Mortality: A Systematic Review and Meta-Analyses of Published Studies. PLoS One.

[4] Algra AM, Rothwell PM (2012). Effects of regular aspirin on long-term cancer incidence and metastasis: a systematic comparison of evidence from observational studies versus randomised trials. The Lancet Oncology.

[5] Rothwell PM (2012). Effect of daily aspirin on risk of cancer metastasis: a study of incident cancers during randomised controlled trials. The Lancet.

[6] Holmes MD (2010). Aspirin intake and survival after breast cancer. Journal of clinical oncology: official journal of the American Society of Clinical Oncology.

[7] Choe KS (2012). Aspirin use and the risk of prostate cancer mortality in men treated with prostatectomy or radiotherapy. Journal of clinical oncology: official journal of the American Society of Clinical Oncology.

[8] Leitzmann MF (2002). Aspirin use in relation to risk of prostate cancer. Cancer Epidemiol Biomarkers Prev.

[9] Ljung R, Sennerstam R, Mattsson F, Auer G, Lagergren J (2014). Anticoagulant medication at time of needle biopsy for breast cancer in relation to risk of lymph node metastasis. International journal of cancer.

[10] Dell’Atti L (2014). Correlation between prolonged use of aspirin and prognostic risk in prostate cancer. Tumori.

[11] Jacobs CD (2014). Aspirin improves outcome in high risk prostate cancer patients treated with radiation therapy. Cancer biology & therapy.

[12] Bradley MC, Black A, Freedman AN, Barron TI (2016). Prediagnostic aspirin use and mortality in women with stage I to III breast cancer: A cohort study in the Prostate, Lung, Colorectal, and Ovarian Cancer Screening Trial. Cancer.

[13] Jonsson F (2013). Low-dose aspirin use and cancer characteristics: a population-based cohort study. British journal of cancer.

[14] Allott EH (2014). Non-steroidal anti-inflammatory drug use, hormone receptor status, and breast cancer-specific mortality in the Carolina Breast Cancer Study. Breast cancer research and treatment.

[15] Sharpe CR (2000). Nested case-control study of the effects of non-steroidal anti-inflammatory drugs on breast cancer risk and stage. British journal of cancer.

[16] Valsecchi ME, Pomerantz SC, Jaslow R, Tester W (2009). Reduced risk of bone metastasis for patients with breast cancer who use COX-2 inhibitors. Clinical breast cancer.

[17] Araujo JL (2016). Prediagnosis aspirin use and outcomes in a prospective cohort of esophageal cancer patients. Therap Adv Gastroenterol.

[18] Barron, T. I., Flahavan, E. M., Sharp, L., Bennett, K. & Visvanathan, K. Recent prediagnostic aspirin use, lymph node involvement, and 5-year mortality in women with stage I-III breast cancer: a nationwide population-based cohort study. *Cancer research* **74**, 4065–4077, doi:10.1158/0008-5472.CAN-13-2679 (2014). 10.1158/0008-5472.CAN-13-2679 PMC4523081 25085874

[19] Menezes, R. J., Swede, H., Niles, R. & Moysich, K. B. Regular use of aspirin and prostate cancer risk (United States). *Cancer causes & control: CCC* **17**, 251–256, doi:10.1007/s10552-005-0450-z (2006). 10.1007/s10552-005-0450-z 16489532

[20] Sansbury LB (2005). Use of nonsteroidal antiinflammatory drugs and risk of colon cancer in a population-based, case-control study of African Americans and Whites. American journal of epidemiology.

[21] Bowers, L. W. *et al*. NSAID use reduces breast cancer recurrence in overweight and obese women: role of prostaglandin-aromatase interactions. *Cancer research* **74**, 4446–4457, doi:10.1158/0008-5472.CAN-13-3603 (2014). 10.1158/0008-5472.CAN-13-3603 PMC12935481 25125682

[22] Forget P (2013). Neutrophil:lymphocyte ratio and intraoperative use of ketorolac or diclofenac are prognostic factors in different cohorts of patients undergoing breast, lung, and kidney cancer surgery. Annals of surgical oncology.

[23] Rothwell PM (2012). Short-term effects of daily aspirin on cancer incidence, mortality, and non-vascular death: analysis of the time course of risks and benefits in 51 randomised controlled trials. Lancet.

[24] Dhillon PK, Kenfield SA, Stampfer MJ, Giovannucci EL (2011). Long-term aspirin use and the risk of total, high-grade, regionally advanced and lethal prostate cancer in a prospective cohort of health professionals, 1988-2006. International journal of cancer.

[25] Kwan, M. L., Habel, L. A., Slattery, M. L. & Caan, B. NSAIDs and breast cancer recurrence in a prospective cohort study. *Cancer causes & control: CCC* **18**, 613–620, doi:10.1007/s10552-007-9003-y (2007). 10.1007/s10552-007-9003-y PMC3461348 17404892

[26] Flossmann E, Rothwell PM (2007). British Doctors Aspirin, T. & the, U. K. T. I. A. A. T. Effect of aspirin on long-term risk of colorectal cancer: consistent evidence from randomised and observational studies. Lancet.

[27] Smith MR (2006). Celecoxib versus placebo for men with prostate cancer and a rising serum prostate-specific antigen after radical prostatectomy and/or radiation therapy. Journal of clinical oncology: official journal of the American Society of Clinical Oncology.

[28] Habel LA, Zhao W, Stanford JL (2002). Daily aspirin use and prostate cancer risk in a large, multiracial cohort in the US. Cancer causes & control: CCC.

[29] Massague J, Obenauf AC (2016). Metastatic colonization by circulating tumour cells. Nature.

[30] Piskounova E (2015). Oxidative stress inhibits distant metastasis by human melanoma cells. Nature.

[31] Baandrup L, Kjaer SK, Olsen JH, Dehlendorff C, Friis S (2015). Low-dose aspirin use and the risk of ovarian cancer in Denmark. Annals of oncology: official journal of the European Society for Medical Oncology.

[32] Macfarlane TV, Murchie P, Watson MC (2015). Aspirin and other non-steroidal anti-inflammatory drug prescriptions and survival after the diagnosis of head and neck and oesophageal cancer. Cancer epidemiology.

[33] McCowan, C., Munro, A. J., Donnan, P. T. & Steele, R. J. Use of aspirin post-diagnosis in a cohort of patients with colorectal cancer and its association with all-cause and colorectal cancer specific mortality. *European journal of cancer* (*Oxford, England: 1990*) **49**, 1049–1057, doi:10.1016/j.ejca.2012.10.024 (2013). 10.1016/j.ejca.2012.10.024 23182687

[34] Algra AM, Rothwell PM (2012). Effects of regular aspirin on long-term cancer incidence and metastasis: a systematic comparison of evidence from observational studies versus randomised trials. Lancet Oncol.

[35] Kinnunen PT (2016). Warfarin use and prostate cancer risk in the Finnish Randomized Study of Screening for Prostate Cancer. Scandinavian journal of urology.

[36] Rothwell PM (2011). Effect of daily aspirin on long-term risk of death due to cancer: analysis of individual patient data from randomised trials. Lancet (London, England).

[37] Liao, L. M. *et al*. Nonsteroidal anti-inflammatory drug use reduces risk of adenocarcinomas of the esophagus and esophagogastric junction in a pooled analysis. *Gastroenterology* **142**, 442–452.e445; quiz e422–443, 10.1053/j.gastro.2011.11.019 (2012). 10.1053/j.gastro.2011.11.019 PMC3488768 22108196

[38] Salinas CA (2010). Use of aspirin and other nonsteroidal antiinflammatory medications in relation to prostate cancer risk. Am J Epidemiol.

[39] Chen K (2014). Co-expression of CD133, CD44v6 and human tissue factor is associated with metastasis and poor prognosis in pancreatic carcinoma. Oncology reports.

[40] Wu Y, Zhou BP (2009). Inflammation: a driving force speeds cancer metastasis. Cell cycle (Georgetown, Tex.).

[41] Hu Y, Yu X, Xu G, Liu S (2016). Metastasis: an early event in cancer progression. Journal of cancer research and clinical oncology.

[42] Husemann Y (2008). Systemic spread is an early step in breast cancer. Cancer cell.

[43] Yuhara, H. *et al*. Is diabetes mellitus an independent risk factor for colon cancer and rectal cancer? *The American journal of gastroenterology* **106**, 1911–1921, quiz 1922, doi:10.1038/ajg.2011.301 (2011). 10.1038/ajg.2011.301 PMC3741453 21912438

[44] Zeng WT, Liu ZH, Li ZY, Zhang M, Cheng YJ (2016). Digoxin Use and Adverse Outcomes in Patients With Atrial Fibrillation. Medicine (Baltimore).

