# Author Correction: NSAIDs Use and Reduced Metastasis in Cancer Patients: results from a meta-analysis

**DOI:** 10.1038/s41598-018-28806-y

**Published:** 2018-07-24

**Authors:** Xiaoping Zhao, Zhi Xu, Haoseng Li

**Affiliations:** 1 Center of Stomatology, Tongji Hospital, Tongji Medical College, Huazhong University of Science and Technology, Wuhan, China; 2 Department of Stomatology, Union Hospital, Tongji Medical College, Huazhong University of Science and Technology, Wuhan, 430022 China

Correction to: *Scientific Reports* 10.1038/s41598-017-01644-0, published online 12 May 2017

In the original version of this Article, Zhi Xu was incorrectly affiliated with ‘Cancer Center, Union Hospital, Tongji Medical College, Huazhong University of Science and Technology, Wuhan, China’. The correct affiliation is listed below.

Department of Stomatology, Union Hospital, Tongji Medical College, Huazhong University of Science and Technology, Wuhan 430022, China.

This error has now been corrected in the PDF and HTML versions of the Article.

