# Supplementary material for: NSAIDs Use and Reduced Metastasis in Cancer Patients: results from a meta-analysis
Source: Sci Rep. 2017 May 12;7:1875. doi: 10.1038/s41598-017-01644-0 (PMC5431951; doi:10.1038/s41598-017-01644-0)
Supplement: Supplementary file 2 — Supplementary Dataset 1 [file 41598_2017_1644_MOESM2_ESM.doc]

# NSAIDs Use and Reduced Metastasis in Cancer Patients: results from a meta-analysis

**Authors**: Xiaoping Zhao ^1^*, Zhi Xu ^2^, Haoseng Li^1^

Table 1

|  |  |  |  | association | | heterogeneity |
| --- | --- | --- | --- | --- | --- | --- |
| study | year | time | cancer | RR(95% CI) | P | *I*^2^ |
| Sharpe^15^ | 2000 | pre | breast | 0.659(0.569-0.764) |  |  |
| Leitzmann^8^ | 2002 | pre | prostate | 0.71(0.38-1.33) |  |  |
| Sansbury^20^ | 2005 | pre | colon | 0.28(0.11-0.7) |  |  |
| Menezes^19^ | 2006 | pre | prostate | 0.7(0.47-1.03) |  |  |
| Rothwell^5^ | 2012 | pre | All solid | 0.64(0.48-0.84) |  |  |
| Jonsson^13^ | 2013 | pre | multi-solid | 0.804(0.718-0.9) |  |  |
| Araujo^17^ | 2016 | pre | esophageal | 3.59(1.08-11.96) |  |  |
| **Sub-total (random mode)** |  |  |  | **0.708(0.586-0.856)** | **0** | **63.40%** |
| Valsecchi^16^ | 2009 | post | breast | 0.12(0.02-0.88) |  |  |
| Holmes^6^ | 2010 | post | breast | 0.502(0.373-0.676)) |  |  |
| Choe^7^ | 2012 | post | prostate | 0.5(0.37-0.68) |  |  |
| Jacobs^11^ | 2014 | post | prostate | 0.293(0.095-0.901) |  |  |
| **Sub-total (random mode)** |  |  |  | **0.484(0.393-0.595)** | **0** | **0** |
| **Overall (random mode)** |  |  |  | **0.623(0.515-0.753)** | **0** | **69.50%** |

pre: pre-diagnosis NSAIDs use

post: post-diagnosis NSAIDs use
