# Supplementary material for: NSAIDs Use and Reduced Metastasis in Cancer Patients: results from a meta-analysis
Source: Sci Rep. 2017 May 12;7:1875. doi: 10.1038/s41598-017-01644-0 (PMC5431951; doi:10.1038/s41598-017-01644-0)
Supplement: Supplementary file 4 — Supplementary Dataset 3 [file 41598_2017_1644_MOESM4_ESM.doc]

# NSAIDs Use and Reduced Metastasis in Cancer Patients: results from a meta-analysis

**Authors**: Xiaoping Zhao ^1^*, Zhi Xu ^2^, Haoseng Li^1^

Table 3

|  |  |  |  | association | | heterogeneity |
| --- | --- | --- | --- | --- | --- | --- |
| study | year | time | cancer | RR(95% CI) | P | *I*^2^ |
| Leitzmann^8^ | 2002 | pre | prostate | 0.71(0.38-1.33) |  |  |
| Menezes^19^ | 2006 | pre | prostate | 0.7(0.47-1.03) |  |  |
| Rothwell^5^ | 2012 | pre | prostate | 0.64(0.27-1.53) |  |  |
| Jonsson^13^ | 2013 | pre | prostate | 0.9(0.8-1) |  |  |
| **Sub-total (fixed mode)** |  |  |  | **0.874(0.787-0.97)** | **0.012** | **0** |
| Choe^7^ | 2012 | post | prostate | 0.5(0.37-0.68) |  |  |
| Jacobs^11^ | 2014 | post | prostate | 0.293(0.095-0.901) |  |  |
| **Sub-total (fixed mode)** |  |  |  | **0.482(0.359-0.647)** | **0** | **0** |
| **Overall (fixed mode)** |  |  |  | **0.817(0.74-0.902)** | **0** | **70.90%** |

pre: pre-diagnosis NSAIDs use

post: post-diagnosis NSAIDs use
