# Supplementary material for: NSAIDs Use and Reduced Metastasis in Cancer Patients: results from a meta-analysis
Source: Sci Rep. 2017 May 12;7:1875. doi: 10.1038/s41598-017-01644-0 (PMC5431951; doi:10.1038/s41598-017-01644-0)
Supplement: Supplementary file 5 — Supplementary Dataset 4 [file 41598_2017_1644_MOESM5_ESM.doc]

# NSAIDs Use and Reduced Metastasis in Cancer Patients: results from a meta-analysis

**Authors**: Xiaoping Zhao ^1^*, Zhi Xu ^2^, Haoseng Li^1^

Table 4

|  |  |  |  | association | | heterogeneity |
| --- | --- | --- | --- | --- | --- | --- |
| study | year | time | cancer | RR(95% CI) | P | *I*^2^ |
| Sharpe^15^ | 2000 | pre | breast | 0.659(0.569-0.764) |  |  |
| Rothwell^5^ | 2012 | pre | breast | 0.5(0.16-1.51) |  |  |
| Jonsson^13^ | 2013 | pre | breast | 0.6(0.5-0.9) |  |  |
| **Sub-total (random mode)** |  |  |  | **0.644(0.565-0.735)** | **0** | **0** |
| Valsecchi^16^ | 2009 | post | breast | 0.12(0.02-0.88) |  |  |
| Holmes^6^ | 2010 | post | breast | 0.502(0.373-0.676) |  |  |
| **Sub-total (random mode)** |  |  |  | **0.485(0.362-0.651)** | **0** | **53.40%** |
| **Overall (random mode)** |  |  |  | **0.615(0.546-0.693)** | **0** | **29.30%** |

pre: pre-diagnosis NSAIDs use

post: post-diagnosis NSAIDs use
