# Supplementary material for: NSAIDs Use and Reduced Metastasis in Cancer Patients: results from a meta-analysis
Source: Sci Rep. 2017 May 12;7:1875. doi: 10.1038/s41598-017-01644-0 (PMC5431951; doi:10.1038/s41598-017-01644-0)
Supplement: Supplementary file 6 — Supplementary Dataset 5 [file 41598_2017_1644_MOESM6_ESM.doc]

# NSAIDs Use and Reduced Metastasis in Cancer Patients: results from a meta-analysis

**Authors**: Xiaoping Zhao ^1^*, Zhi Xu ^2^, Haoseng Li^1^

Table 5

|  |  |  |  | association | | heterogeneity |
| --- | --- | --- | --- | --- | --- | --- |
| study | year | time | cancer | RR(95% CI) | P | *I*^2^ |
| Sansbury^20^ | 2005 | pre | colorectal | 0.28(0.11-0.7) |  |  |
| Rothwell^5^ | 2012 | pre | colorectal | 0.36(0.18-0.74) |  |  |
| Jonsson^13^ | 2013 | pre | colorectal | 0.8(0.7-0.9) |  |  |
| **Total (random mode)** |  |  |  | **0.477(0.23-0.988)** | **0.046** | **0.787** |
| Rothwell^5^ | 2012 | pre | lung | 0.55(0.26-1.17) |  |  |
| Jonsson^13^ | 2013 | pre | lung | 0.8(0.7-0.9) |  |  |
| **Total (random mode)** |  |  |  | **0.792(0.7-0.896)** | **0** | **0** |

pre: pre-diagnosis NSAIDs use
