# Supplementary material for: NSAIDs Use and Reduced Metastasis in Cancer Patients: results from a meta-analysis
Source: Sci Rep. 2017 May 12;7:1875. doi: 10.1038/s41598-017-01644-0 (PMC5431951; doi:10.1038/s41598-017-01644-0)
Supplement: Supplementary file 7 — Supplementary Dataset 6 [file 41598_2017_1644_MOESM7_ESM.doc]

# NSAIDs Use and Reduced Metastasis in Cancer Patients: results from a meta-analysis

**Authors**: Xiaoping Zhao ^1^*, Zhi Xu ^2^, Haoseng Li^1^

Table 6

|  |  |  |  | association | | heterogeneity |
| --- | --- | --- | --- | --- | --- | --- |
| study | year | time | cancer | RR(95% CI) | P | *I*^2^ |
| Jonsson^13^ | 2013 | pre | multi-solid | 0.955(0.907-1.006) |  |  |
| Allott^14^ | 2014 | pre | prostate | 1.1(0.824-1.467) |  |  |
| Atti^10^ | 2014 | pre | breast | 0.574(0.31-1.063) |  |  |
| Ljung^9^ | 2014 | pre | breast | 0.94(0.87-1.03) |  |  |
| Barron^18^ | 2014 | pre | breast | 0.89(0.81-0.97) |  |  |
| Bradley^12^ | 2016 | pre | breast | 1.04(0.92-1.17) |  |  |
| **Overall (fixed mode)** |  |  |  | **0.949(0.914-0.985)** | **0.006** | **0.363** |

pre: pre-diagnosis NSAIDs use
