# Supplementary material for: NSAIDs Use and Reduced Metastasis in Cancer Patients: results from a meta-analysis
Source: Sci Rep. 2017 May 12;7:1875. doi: 10.1038/s41598-017-01644-0 (PMC5431951; doi:10.1038/s41598-017-01644-0)
Supplement: Supplementary file 9 — Table legends [file 41598_2017_1644_MOESM9_ESM.pdf]

NSAIDs Use and Reduced Metastasis in Cancer Patients: results from a meta-analysis

Authors: Xiaoping Zhao [1]*, Zhi Xu [2], Haoseng Li[1]

Table legend

Supplementary Dataset 1: Reduced relative risks of distant metastasis development associated with NSAIDs use.

Supplementary Dataset 2: After removal of James L. Araujo's study, the heterogeneity among studies on pre-diagnosis use of NSAIDs decreased, and the inhibition by pre-diagnostic NSAID use slightly strengthened.

Supplementary Dataset 3: Reduced relative risks of prostate cancer metastasis development associated with NSAID use.

Supplementary Dataset 4: Reduced relative risks of breast cancer metastasis development associated with NSAID use.

Supplementary Dataset 5: Reduced relative risks of colorectal and lung cancer metastasis development associated with NSAID use.

Supplementary Dataset 6: The week association between cancer lymph node metastasis and NSAIDs use.

Supplementary Dataset 7: The week association between breast and prostate cancer lymph node metastasis and NSAIDs use.

Supplemental table: decision of the gathered 24 studies.

Supplementary Info File: characteristics of the included studies.

Quality: included studies' quality was evaluated according to Newcastle–Ottawa Scale (NOS) criteria.
