# Supplementary material for: NSAIDs Use and Reduced Metastasis in Cancer Patients: results from a meta-analysis
Source: Sci Rep. 2017 May 12;7:1875. doi: 10.1038/s41598-017-01644-0 (PMC5431951; doi:10.1038/s41598-017-01644-0)
Supplement: Supplementary file 10 — Supplementary Dataset 8 [file 41598_2017_1644_MOESM10_ESM.doc]

Table. decision of the gathered studies

| Num. | Study | Choice | Reason |
| --- | --- | --- | --- |
| 1 | Smith，2006^1^ | removed | Recurrence contains subsequent local, regional, and distant disease |
| 2 | Kwan, 2007^2^ | removed | Recurrence contains subsequent local, regional, and distant disease |
| 3 | Flossmann, 2007^3^ | removed | Not the complete study |
| 4 | Forget, 2013^4^ | removed | Intraoperative Use of NSAIDs |
| 5 | Bowers, 2014^5^ | removed | Recurrence contains subsequent local, regional, and distant disease |
| 6 | Dhillon, 2011^6^ | removed | Lymph node or distant metastasis was mixed with other data |
| 7 | Habel, 2002^7^ | removed | Recurrence contains Regional and distant disease |
| 8 | Rothwell, 2012^8^ | removed | Not the complete study |
| 9 | Ljung, 2014^9^ | accepted | Meeting the acceptable criteria |
| 10 | Rothwell, 2012^10^ | accepted | Meeting the acceptable criteria |
| 11 | Jacobs, 2014^11^ | accepted | Meeting the acceptable criteria |
| 12 | Atti, 2014^12^ | accepted | Meeting the acceptable criteria |
| 13 | Choe, 2012^13^ | accepted | Meeting the acceptable criteria |
| 14 | Holmes, 2010^14^ | accepted | Meeting the acceptable criteria |
| 15 | Leitzmann, 2002^15^ | accepted | Meeting the acceptable criteria |
| 16 | Valsecchi, 2009^16^ | accepted | Meeting the acceptable criteria |
| 17 | Barron, 2014^17^ | accepted | Meeting the acceptable criteria |
| 18 | Sansbury, 2005^18^ | accepted | Meeting the acceptable criteria |
| 19 | Jonsson, 2013^19^ | accepted | Meeting the acceptable criteria |
| 20 | Menezes, 2006^20^ | accepted | Meeting the acceptable criteria |
| 21 | Sharpe, 2000^21^ | accepted | Meeting the acceptable criteria |
| 22 | Araujo, 2016^22^ | accepted | Meeting the acceptable criteria |
| 23 | Bradley, 2016^23^ | accepted | Meeting the acceptable criteria |
| 24 | Allott, 2014^24^ | accepted | Meeting the acceptable criteria |

**Reference**

1 Smith, M. R. *et al.* Celecoxib versus placebo for men with prostate cancer and a rising serum prostate-specific antigen after radical prostatectomy and/or radiation therapy. *Journal of clinical oncology : official journal of the American Society of Clinical Oncology* **24**, 2723-2728, doi:10.1200/JCO.2005.03.7804 (2006).

2 Kwan, M. L., Habel, L. A., Slattery, M. L. & Caan, B. NSAIDs and breast cancer recurrence in a prospective cohort study. *Cancer causes & control : CCC* **18**, 613-620, doi:10.1007/s10552-007-9003-y (2007).

3 Flossmann, E., Rothwell, P. M., British Doctors Aspirin, T. & the, U. K. T. I. A. A. T. Effect of aspirin on long-term risk of colorectal cancer: consistent evidence from randomised and observational studies. *Lancet* **369**, 1603-1613, doi:10.1016/S0140-6736(07)60747-8 (2007).

4 Forget, P. *et al.* Neutrophil:lymphocyte ratio and intraoperative use of ketorolac or diclofenac are prognostic factors in different cohorts of patients undergoing breast, lung, and kidney cancer surgery. *Annals of surgical oncology* **20 Suppl 3**, S650-660, doi:10.1245/s10434-013-3136-x (2013).

5 Bowers, L. W. *et al.* NSAID use reduces breast cancer recurrence in overweight and obese women: role of prostaglandin-aromatase interactions. *Cancer research* **74**, 4446-4457, doi:10.1158/0008-5472.CAN-13-3603 (2014).

6 Dhillon, P. K., Kenfield, S. A., Stampfer, M. J. & Giovannucci, E. L. Long-term aspirin use and the risk of total, high-grade, regionally advanced and lethal prostate cancer in a prospective cohort of health professionals, 1988-2006. *International journal of cancer* **128**, 2444-2452, doi:10.1002/ijc.25811 (2011).

7 Habel, L. A., Zhao, W. & Stanford, J. L. Daily aspirin use and prostate cancer risk in a large, multiracial cohort in the US. *Cancer causes & control : CCC* **13**, 427-434 (2002).

8 Rothwell, P. M. *et al.* Short-term effects of daily aspirin on cancer incidence, mortality, and non-vascular death: analysis of the time course of risks and benefits in 51 randomised controlled trials. *Lancet* **379**, 1602-1612, doi:10.1016/S0140-6736(11)61720-0 (2012).

9 Ljung, R., Sennerstam, R., Mattsson, F., Auer, G. & Lagergren, J. Anticoagulant medication at time of needle biopsy for breast cancer in relation to risk of lymph node metastasis. *International journal of cancer* **135**, 238-241, doi:10.1002/ijc.28671 (2014).

10 Rothwell, P. M. *et al.* Effect of daily aspirin on risk of cancer metastasis: a study of incident cancers during randomised controlled trials. *The Lancet* **379**, 1591-1601, doi:10.1016/s0140-6736(12)60209-8 (2012).

11 Jacobs, C. D. *et al.* Aspirin improves outcome in high risk prostate cancer patients treated with radiation therapy. *Cancer biology & therapy* **15**, 699-706, doi:10.4161/cbt.28554 (2014).

12 Dell'Atti, L. Correlation between prolonged use of aspirin and prognostic risk in prostate cancer. *Tumori* **100**, 486-490, doi:10.1700/1660.18156 (2014).

13 Choe, K. S. *et al.* Aspirin use and the risk of prostate cancer mortality in men treated with prostatectomy or radiotherapy. *Journal of clinical oncology : official journal of the American Society of Clinical Oncology* **30**, 3540-3544, doi:10.1200/JCO.2011.41.0308 (2012).

14 Holmes, M. D. *et al.* Aspirin intake and survival after breast cancer. *Journal of clinical oncology : official journal of the American Society of Clinical Oncology* **28**, 1467-1472, doi:10.1200/JCO.2009.22.7918 (2010).

15 Leitzmann, M. F. *et al.* Aspirin use in relation to risk of prostate cancer. *Cancer Epidemiol Biomarkers Prev* **11**, 1108-1111 (2002).

16 Valsecchi, M. E., Pomerantz, S. C., Jaslow, R. & Tester, W. Reduced risk of bone metastasis for patients with breast cancer who use COX-2 inhibitors. *Clinical breast cancer* **9**, 225-230, doi:10.3816/CBC.2009.n.038 (2009).

17 Barron, T. I., Flahavan, E. M., Sharp, L., Bennett, K. & Visvanathan, K. Recent prediagnostic aspirin use, lymph node involvement, and 5-year mortality in women with stage I-III breast cancer: a nationwide population-based cohort study. *Cancer research* **74**, 4065-4077, doi:10.1158/0008-5472.CAN-13-2679 (2014).

18 Sansbury, L. B. *et al.* Use of nonsteroidal antiinflammatory drugs and risk of colon cancer in a population-based, case-control study of African Americans and Whites. *American journal of epidemiology* **162**, 548-558, doi:10.1093/aje/kwi248 (2005).

19 Jonsson, F. *et al.* Low-dose aspirin use and cancer characteristics: a population-based cohort study. *British journal of cancer* **109**, 1921-1925, doi:10.1038/bjc.2013.411 (2013).

20 Menezes, R. J., Swede, H., Niles, R. & Moysich, K. B. Regular use of aspirin and prostate cancer risk (United States). *Cancer causes & control : CCC* **17**, 251-256, doi:10.1007/s10552-005-0450-z (2006).

21 Sharpe, C. R. *et al.* Nested case-control study of the effects of non-steroidal anti-inflammatory drugs on breast cancer risk and stage. *British journal of cancer* **83**, 112-120, doi:10.1054/bjoc.2000.1119 (2000).

22 Araujo, J. L. *et al.* Prediagnosis aspirin use and outcomes in a prospective cohort of esophageal cancer patients. *Therap Adv Gastroenterol* **9**, 806-814, doi:10.1177/1756283X16657985 (2016).

23 Bradley, M. C., Black, A., Freedman, A. N. & Barron, T. I. Prediagnostic aspirin use and mortality in women with stage I to III breast cancer: A cohort study in the Prostate, Lung, Colorectal, and Ovarian Cancer Screening Trial. *Cancer* **122**, 2067-2075, doi:10.1002/cncr.30004 (2016).

24 Allott, E. H. *et al.* Non-steroidal anti-inflammatory drug use, hormone receptor status, and breast cancer-specific mortality in the Carolina Breast Cancer Study. *Breast cancer research and treatment* **147**, 415-421, doi:10.1007/s10549-014-3099-z (2014).
